# Quantification of Cytokeratin 5 mRNA Expression in the Circulation of Healthy Human Subjects and after Lung Transplantation

**DOI:** 10.1371/journal.pone.0005925

**Published:** 2009-06-16

**Authors:** Derek W. Nickerson, Angela P. Presson, Stephen S. Weigt, Aric L. Gregson, John A. Belperio, Brigitte N. Gomperts

**Affiliations:** 1 Mattel Children's Hospital at UCLA, Division of Hematology Oncology, Department of Pediatrics, David Geffen School of Medicine at UCLA, Los Angeles, California, United States of America; 2 Department of Biostatistics, University of California Los Angeles, Los Angeles, California, United States of America; 3 Division of Pulmonary and Critical Care, Department of Medicine, David Geffen School of Medicine at UCLA, Los Angeles, California, United States of America; 4 Division of Infectious Diseases, Department of Medicine, David Geffen School of Medicine at UCLA, Los Angeles, California, United States of America; University of Birmingham, United Kingdom

## Abstract

**Background:**

Circulating epithelial progenitor cells are important for repair of the airway epithelium in a mouse model of tracheal transplantation. We therefore hypothesized that circulating epithelial progenitor cells would also be present in normal human subjects and could be important for repair of the airway after lung injury. As lung transplantation is associated with lung injury, which is severe early on and exacerbated during episodes of infection and rejection, we hypothesized that circulating epithelial progenitor cell levels could predict clinical outcome following lung transplantation.

**Methodology/Principal Findings:**

Quantitative Real Time PCR was performed to determine peripheral blood mRNA levels of cytokeratin 5, a previously characterized marker of circulating epithelial progenitor cells. Cytokeratin 5 levels were evaluated in healthy human subjects, in lung transplant recipients immediately post-transplant and serially thereafter, and in heart transplant recipients. All normal human subjects examined expressed cytokeratin 5 in their buffy coat in amounts that were not significantly influenced by age or gender. There was a profound, statistically significant decrease in cytokeratin 5 mRNA expression levels in lung transplant patients compared to healthy human subjects (p = 3.1×10^−13^) and to heart transplant recipients. There was a moderate negative correlation between improved circulating cytokeratin 5 mRNA levels in lung transplant recipients with recovering lung function, as measured by improved FEV1 values (rho = −0.39).

**Conclusions/Significance:**

Levels of cytokeratin 5 mRNA, a proxy marker for circulating epithelial progenitor cells, inversely correlated with disease status in lung transplant recipients. It may therefore serve as a biomarker of the clinical outcome of lung transplant patients and potentially other patients with airway injury.

## Introduction

The proximal airway epithelium is in contact with the environment and, as such, is at constant jeopardy from environmental injury. An efficient mechanism for airway repair is therefore essential to protect the host. Our current understanding of proximal airway repair is that a progenitor cell pool is located in the submucosal glands and submucosal gland ducts that are capable of self renewal and of differentiating in to the proximal airway subtypes e.g. mucus and ciliated cells [1], [2], [3], [4], [5]. These progenitor cells express the immature cytokeratins (CK) CK5 and CK14 and move up the submucosal gland ducts to form the basal layer of the pseudostratified columnar epithelium of the proximal airway. From there the basal cells lose CK5/14 and gain more mature cytokeratins e.g. CK8/18 as they differentiate and move apically.

We have shown the presence of circulating CK5 expressing cells that contributed to airway repair in a mouse model of ischemic injury and proximal airway repair [6]. We used FACS analysis to show the presence of CK5 expressing cells in the bone marrow and circulation of mice [6]. The identification of circulating epithelial cells that contribute to airway repair represents a controversial paradigm shift in the current concept of airway repair and regeneration after injury. The overall aims of this study were to determine whether CK5 mRNA expression could be quantified in the circulation of normal human subjects and to determine whether CK5 mRNA levels would be altered with severe airway disease, such as in lung transplant patients with end stage lung disease. We also hypothesized that CK5 mRNA expression levels would increase as patients recovered post lung transplant and could function as a clinical biomarker of airway disease.

## Results

### Detection of CK5 in the Circulation of Normal Human Subjects and Patients by Conventional PCR

**Figure 1:**
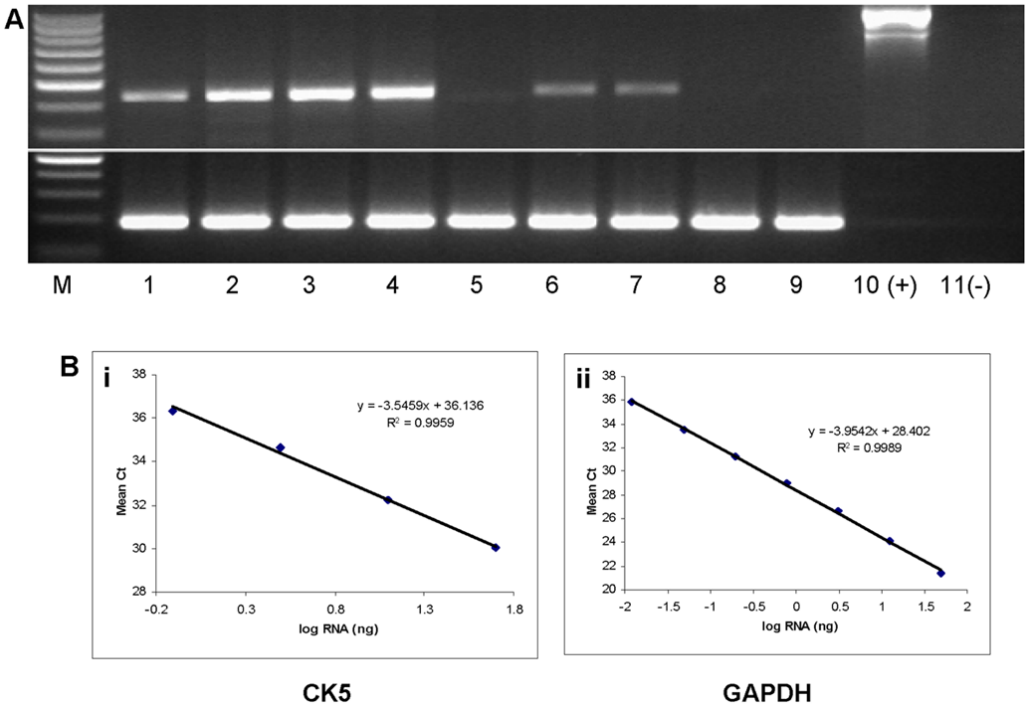
A. PCR for CK5 mRNA from the circulation of healthy volunteers and lung transplant patients. The top panel shows the expected 439 bp fragment for CK5 using cDNA as template in healthy volunteers (Lanes 1–4) and CK5 mRNA expression from a representative group of patients post lung transplantation (Lanes 5–9). CK5 mRNA expression was not found in PCR Lanes 5, 8 and 9 and neither was CK5 mRNA expression detectible by quantitative real-time PCR in these samples. Lane 10 represents the positive control, which consists of a bacterial artificial chromosome (BAC) containing 170 kb of genomic sequence surrounding the CK5 locus as template, which is why the band is a larger size. Lane 11 is the negative control without cDNA template. The bottom panel shows PCR amplification of GAPDH from the same templates. B. Standard curve of real-time PCR amplification of CK5 and GAPDH. The log quantity of RNA is plotted against the mean threshold cycle (Ct), measured in triplicate. Slope and intercept are represented in the equations of the regression lines, along with the regression coefficient.

We performed conventional PCR on cDNA obtained from the blood of normal human subjects and detected message for CK5 in all normal human subjects examined. PCR on lung transplant patient cDNA samples from the buffy coat revealed the presence of mRNA for CK5 in only some of the lung transplant patients. PCR with GAPDH primers was used to confirm the integrity of the cDNA (Figure 1A).

### Validation of the Quantitative Real Time PCR Assay for CK5

The multiplex Quantitative Real Time PCR assay was first performed with varying concentrations of primer and then probe to determine the optimal concentration of primers and probe for the assay (Table 1). The concentration at which, for both primers and probe, the ΔCt and ΔRn did not change with increasing concentrations was selected. Thus primer concentrations of 600 nm and probe concentration of 250 nm were chosen. Then a standard curve consisting of serial dilutions of cDNA was performed with the determined CK5 primer and probe concentrations to determine the linear range of the assay. The triplicate reproducibility was noted to start to fall off the curve at threshold cycle (Ct) values greater than 36 (Figure 1Bi). The linear range of the GAPDH assay is more extensive than the CK5 assay because of the abundance of GAPDH expression (Figure 1Bii).

**Table 1:** Optimization of the Assay for Primers and Probes.

[Primers (ea)] (nM)	[Probe] (nM)	Mean CK5 Ct	CK5 Ct SE	Mean GAPDH Ct
50	250	37.414	1.073	21.894
100	250	31.479	0.282	21.851
200	250	27.62	0.151	21.846
400	250	26.294	0.098	21.754
600	250	26.224	0.175	21.62
900	250	25.955	0.139	21.623
[Primers (ea)] (nM)	[Probe] (nM)	Mean CK5 Ct	CK5 Ct SE	Mean GAPDH Ct
600	50	30.085	0.139	21.651
600	100	29.278	0.078	21.752
600	150	28.815	0.083	21.825
600	200	28.59	0.71	21.9
600	250	28.405	0.023	21.97

The Quantitative Real Time PCR assay was first performed with varying concentrations of primer and then probe to determine the optimal concentration of primers and probe for the assay. The concentration at which, for both primers and probe, the ΔCt and ΔRn did not change with increasing concentrations was selected. Thus primer concentrations of 600 nM and probe concentration of 250 nM were chosen.

### Quantification of the CK5 mRNA in Normal Human Subjects

**Figure 2:**
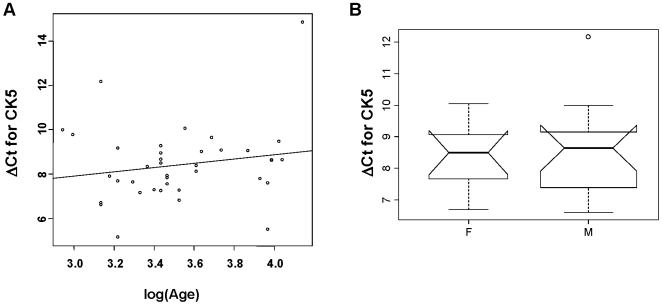
A. Quantitative real-time PCR of CK5 plotted against age of normal human subjects. A scatter plot of data shows no significant difference between the log of the age and CK5 mRNA expression levels in all 38 normal human subjects examined (p = 0.273). B. Quantitative real-time PCR expression of CK5 does not differ with gender. A box plot demonstrates the similarity in CK5 levels in male and female normal human subjects (p = 0.84).

The triplicate Ct values for each sample were averaged resulting in mean Ct values for both CK5 and GAPDH. The CK5 Ct values were then standardized to the housekeeping gene by taking the difference: ΔCt = Ct[CK5]−Ct[GAPDH]. Most of the CK5 quantitative real-time PCR data were analyzed using the ΔCt variable, as it was approximately normally distributed in some sub groups of data. Kendall rank correlation was used to test for a ΔCt relationship with age in the control samples and an exact Wilcoxon rank sum test was used to compare ΔCt distributions between male and female control samples. Both tests yielded non-significant results (p-value = 0.24 and 0.93, respectively). A scatterplot of ΔCt vs. log of age is shown in Figure 2A. A boxplot of the sex-specific ΔCt values shows that there was no significant difference between males and females in our control data (Figure 2B).

### Longitudinal study of CK5 mRNA in Lung Transplant Patients

We followed CK5 levels in a cohort of 23 lung transplant patients, ranging in age from 31–79 years that received transplants at the David Geffen School of Medicine at UCLA between February 2006 and March 2007. All patients had end stage lung disease at the time of transplant. About 30% of patients were transplanted for chronic obstructive pulmonary disease (COPD), about 30% for idiopathic pulmonary fibrosis (IPF) and the remaining for a variety of diagnoses. Most patients had 2–4 appointments within the year following their transplant where blood samples were drawn and their pulmonary function was tested. Table 2 details the lung transplant patient demographics.

**Table 2:** Lung transplant patient demographics.

Patient #	Age (yr)	Gender	Diagnosis	Single/Double Lung
1	58	M	COPD	Double
2	58	M	COPD	Double
3	64	F	COPD	Double
4	62	M	PCH	Double
5	61	M	COPD	Double
6	47	F	sarcoid	Double
7	60	F	COPD	Double
8	57	M	IPF	Double
9	31	M	scleroderma	Double
10	45	F	PHTN	Double
11	59	M	IPF	Double
12	63	F	COPD	Single
13	55	F	ILD	Double
14	60	M	PHTN	Double
15	53	M	ILD	Double
16	63	F	IPF	Single
17	55	F	COPD	Double
18	66	F	COPD	Single
19	56	F	IPF	Double
20	33	M	scleroderma	Double
21	79	M	IPF	Single
22	70	M	IPF	Single
23	62	M	IPF	Double

COPD = chronic obstructive pulmonary disease; IPF = idiopathic pulmonary fibrosis; PHTN = pulmonary hypertension; ILD = interstitial lung disease; PCH = Pulmonary capillary hemangiomatosis.

**Figure 3:**
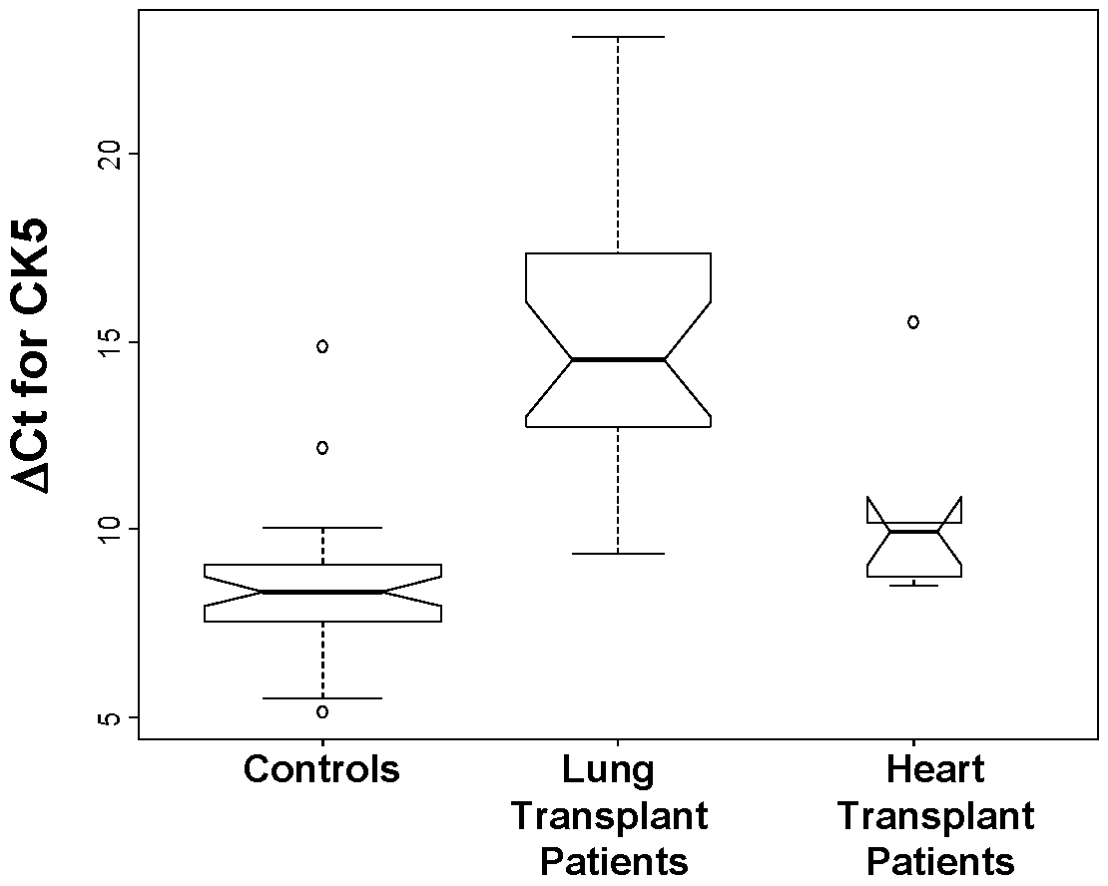
Quantitative real-time PCR of CK5 expression in normal human subjects compared to lung transplant patients and heart transplant patients. A box plot demonstrates the differences between CK5 values in normal human subjects compared to lung transplant patients (p = 3.1×10^−13^). A further comparison is made between CK5 mRNA expression in the circulation of heart transplant patients (n = 6) and lung transplant patients (p = 0.004).

We found a 75-fold decrease in CK5 mRNA expression in the cohort of lung transplant patient samples tested post-transplant in comparison to healthy control subjects with 95% CI (33.36, 204.9) and p-value of 3.1×10^−13^ by the Wilcoxon rank sum test. This result is highly significant even after correcting for the multiple testing per patient, which would conservatively suggest a 0.005 significance level (for approximately 10 tests). Figure 3 shows a boxplot comparison of ΔCt values between cases and controls.

All patients in the cohort were alive at more than one year post transplant, and to date none have developed bronchiolitis obliterans. No correlation in CK5 expression was found in patients with episodes of rejection or infection, although the study is not sufficiently powered to address this question. No significant difference in CK5 expression was found between patients with COPD compared to other airway diseases post-lung transplantation (p-value = 0.106). There was also no significant difference between single and double lung transplant recipients. However, only 5 of the 23 patients received a single lung transplant.

**Figure 4:**
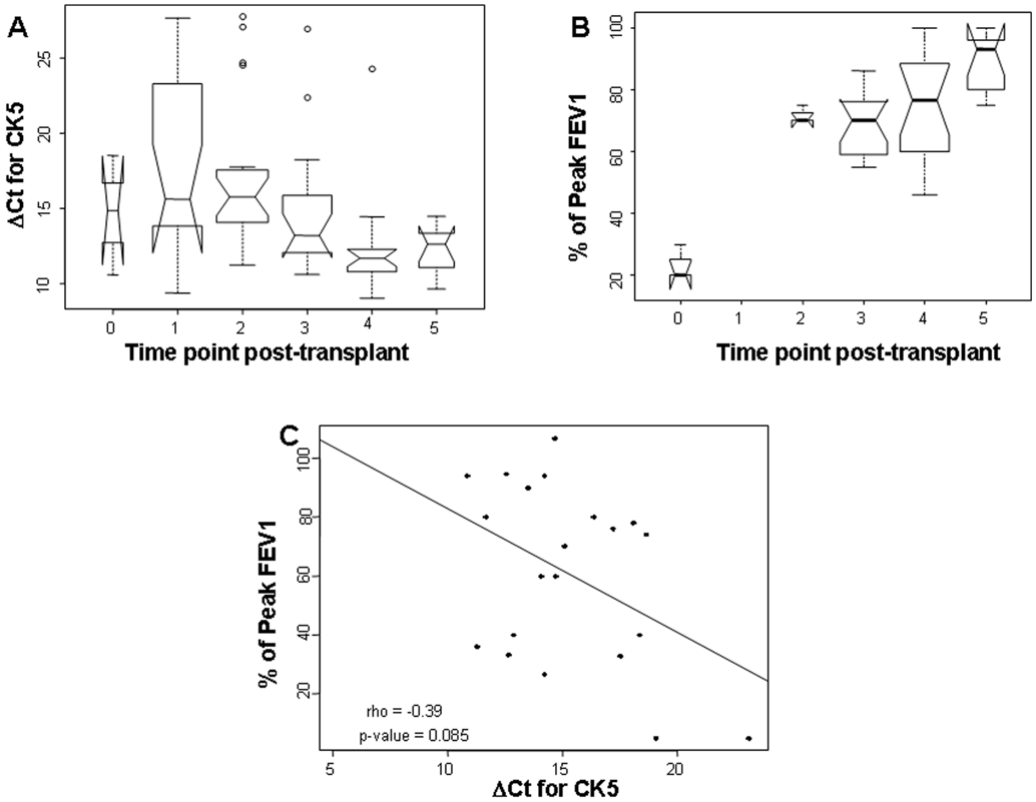
A. Quantitative real-time PCR of CK5 expression in lung transplant patients correlated with time post-transplant. This box plot shows the increase in CK5 mRNA expression (decrease in ΔCt for CK5) with time post transplant. The X-axis represents set time points post transplant (0 = time of transplant; 1 = 1day post-transplant (PT); 2 = 1week PT; 3 = 1month PT; 4 = 3months PT; 5 = 6months PT). B. Percentage decrease in FEV1 in lung transplant patients correlated with time post-transplant. Box plot of FEV1 on the Y-axis with 0.0 being 100% FEV1 compared to time post transplant on the X-axis (0 = time of transplant; 1 = 1day PT; 2 = 1week PT; 3 = 1month PT; 4 = 3months PT; 5 = 6months PT). C. Quantitative real-time PCR of CK5 expression in lung transplant patients correlated with percentage decrease in FEV1 post-transplant. There is a moderate negative correlation between average FEV1 and average ΔCt values of −0.39. However, this does not reach statistical significance with a p-value = 0.085.

We then examined patient outcomes by correlating their CK5 levels with time post-transplantation. CK5 mRNA expression was found to increase from the immediate post transplant period (within the first week when the donor graft has significant ischemic and reperfusion injury), to the latest available time point (typically 3–6 months post transplant) with a 9.2 fold increase in CK5 expression from the first CK5 level measured post-transplant to the last CK5 level post-transplant with 95% CI (2.42, 40.6) and a p-value of 1.4×10^−5^ for the correlation of CK5 mRNA expression and time post-transplant (Figure 4A). As expected, we also found a highly significant correlation between improvement in FEV1 and time post-transplantation (p-value = 4.7×10^−5^) (Figure 4B). There is a moderate negative correlation between average FEV1 and average ΔCt values per patient of −0.39. However, this did not reach statistical significance with a p-value = 0.085 (Figure 4C).

In order to determine whether the reduction in circulating CK5 expression was specific to lung transplant patients, we examined CK5 expression in the circulation of heart transplant patients. We found a significant difference between the CK5 expression levels in heart transplant compared to lung transplant patients, with the heart transplant patients having levels of CK5 expression in the circulation that were significantly greater than the lung transplant patients (p-value = 0.004). There was an approximately 20.6 fold decrease in CK5 mRNA expression in lung transplant patients as compared to heart transplant patients (95% CI: 4.24, 191) (Figure 3). However, there was a trend for heart transplant patients to express less CK5 in circulation than normal human subjects (p-value = 0.05).

## Discussion

Building on our previous studies on CK5 expressing circulating epithelial progenitor cells in the circulation, our present findings demonstrate the presence of CK5-positive cells in circulation in normal human subjects[6]. Furthermore, they correlate the levels of CK5 in circulation with lung status post transplant. The significant reduction in CK5 expressing cells in the circulation of lung transplant patients argues that there may be a role for circulating epithelial progenitor cells in normal airway repair. This is further emphasized by the correlation that was found between an increase in circulating epithelial progenitor cells and an improvement in lung function after transplantation. Together, these results are consistent with the view that circulating epithelial progenitor cells may be critical for normal airway repair and that a lack of circulating epithelial progenitor cells may be associated with airway disease.

One limitation of the patient studies is the inability to determine the origin of the circulating epithelial progenitor cells. Our results suggest that the origin of CK5 expressing cells in circulation may be from the bone marrow, the thymus, or may be derived from the lung tissue itself, or may be derived from all of these compartments. If the origin of the circulating epithelial progenitor cells is the bone marrow then it is possible that the immunosuppression that the lung transplant patients are on could be responsible for the decrease in the expression of CK5. We therefore analyzed the expression of CK5 in the circulation of heart transplant patients who are also on immunosuppression but do not have any major airway epithelial injury. We found a significant difference between the amount of circulating epithelial progenitor cells in heart transplant patients and lung transplant patients, which suggests that circulating epithelial progenitor cells are specific for airway repair. We also found a trend towards heart transplant patients having less circulating epithelial progenitor cells than normal human subjects. The heart transplant group were on lower doses of immunosuppression than the lung transplant patients, which suggests that immunosuppression may be playing a role in the reduction of circulating epithelial progenitor cells found in transplant patients. It is also possible that the differences in CK5 expression in circulation may result from changes in vascular permeability or altered blood supply to the lung allografts.

We did not find a difference in CK5 mRNA expression in normal human subjects with increasing age and this might be expected as normal repair and regeneration decreases with age. However, there are likely many variables in addition to age that contribute to overall repair of the airway. CK5 expressing cells are also found as basal cells in other complicated epithelia, most notably skin and prostate. We would therefore have predicted that gender differences might be seen, although this was not the case in our control samples. However, as benign prostatic hypertrophy is associated with advancing age, it will be important in the future to examine CK5 levels in older normal human subjects to determine if there is a gender difference in this group.

Previous studies on the engraftment of bone marrow-derived epithelial cells in the distal airway after lung injury have shown conflicting results [7], [8], [9], [10], [11], [12]. Our results demonstrated that circulating epithelial cells are present in the circulation of all normal human subjects examined. However, flow cytometry analysis of CK5 expressing cells in circulation are required to confirm this. Our experiments were performed retrospectively on frozen RNA samples and therefore flow cytometry for CK5 could not be performed. Our studies were not designed to assess the magnitude of engraftment of the circulating epithelial cells in the airway. However, the statistically significant difference between lung transplant patients and normal human subjects with regard to their CK5 mRNA levels suggested that circulating epithelial cells may be important in normal airway repair. In addition, the correlation of the increase in CK5 levels with the improvement in pulmonary function testing post-transplantation suggests that CK5 mRNA levels in circulation could be used as a biomarker of airway repair. By implication, it would appear that circulating epithelial progenitor cells most likely do play an important role in airway repair, although whether this is a direct of indirect effect still needs to be established. Ultimately, determining whether CK5 mRNA levels can be linked to the development of either acute or chronic rejection and/or infection is critical and is an area of active investigation in the lab.

In summary, CK5 expressing circulating epithelial progenitor cells are present in circulation in normal human subjects and can be quantified with the real time PCR assay that we have established. Furthermore, the circulating epithelial progenitor cells are significantly reduced immediately after lung transplantation, and then increase with time as lung function improves. We suggest that circulating epithelial progenitor cells may play an important role in airway repair after lung transplantation. In addition, circulating epithelial progenitor cells may be used as a biomarker of airway repair.

## Methods

### Selection and description of participants

#### Ethics statement

The research was approved by the UCLA Institutional Review Board (IRB). IRB approval and informed written consent were obtained from all patients and normal human subjects examined. Data were analyzed anonymously.

Patients that were part of this study were patients at the University of California Los Angeles, and received lung transplants between 2005 and 2006. The patients in the study were placed on standard pre- and post-lung transplant immunosuppression and antimicrobials, as per standard of care. Only patients ≥ 18 years of age were included, as there is no pediatric lung transplant program at UCLA, otherwise all lung transplant recipients at UCLA were included.

Exclusion criteria included individuals involved in a clinical research trial utilizing an investigational therapy, pregnant women, lactating women, or women of childbearing age not willing to take precautions to avoid becoming pregnant during the study.

Subjects underwent peripheral blood collection just prior to lung transplantation. At 24 hours post lung transplant all recipients had their peripheral blood collected and then underwent a bronchoscopy with transbronchial biopsy. In addition, both peripheral blood and bronchoscopy with transbronchial biopsy were performed at 1week, 4 weeks, 3 months and 6 months post-transplantation and also when clinically indicated to evaluate for infection and/or rejection. We reported FEV1 as a percentage of the patient's best FEV1 post-transplant. Therefore, 100% FEV1 is the best FEV1 measurement obtained for a particular patient. The best FEV1 measurement typically occurred after the first 6 months post-transplant.

### Blood samples

Venous blood samples were obtained from 23 lung transplant patients (aged 31–79 years) and 28 healthy volunteers (aged 19–54 years). 3 of the healthy volunteers smoked on a daily basis. None of the healthy volunteers reported any health problems. Blood samples were drawn from 6 heart transplant patients. All samples were collected in EDTA containing tubes. In the volunteer group, two 5 ml samples were collected and the first was discarded. This step was performed to eliminate possible contamination with epithelial cells from the epidermis during venipuncture. In the heart transplant group, samples were collected from central venous catheters. Not all of the patients had all time points measured, but 65% of the patients had at least 4 measurements and 17% of the patients had 1–2 measurements. We had only 3 patients with a pre-transplant measurement.

### RNA extraction and cDNA synthesis

#### Lung Transplant Patients

Total RNA was isolated from buffy coat. Initially, buffy coat was collected from fresh whole blood following a 10 minute spin at 450× g. One (1) ml TRIzol Reagent (Invitrogen, Carlsbad, CA) and 0.2 volume chloroform was added per 5×10^6^ cells and the lysates were centrifuged at 12,000× g. RNA was collected in the aqueous phase. The RNA was then precipitated in isopropanol and washed with 70% ethanol. The resulting pellet was resuspended in 100 µl nuclease free water and further purified using the RNeasy Mini Kit (Qiagen, Valencia, CA) with on-column DNA digestion using the RNase-free DNase kit (Qiagen) as per the manufacturer's instructions.

#### Volunteers and Heart Transplant Patients

Total RNA was extracted from a 1.5 ml aliquot of fresh whole blood using the RNeasy Blood Mini Kit (Qiagen). Manufacturer's instructions were followed except that following lysis of red blood cells, leukocytes were given one additional wash with buffer EL (Qiagen) to ensure removal of erythrocytes and any potential RT-PCR inhibitors. Samples were all treated on-column with the RNase-free DNase kit (Qiagen).

RNA concentration was measured in a Qubit fluorometer (Invitrogen). 1 µg of isolated total RNA was reversed transcribed with oligo-dT primers using the Taqman Reverse Transcription Reagents kit (Applied Biosystems N808-0234) in a final volume of 100 µl following the manufacturer's instructions. Some volunteer whole blood was also processed as described above for the Lung Transplant Patients and no difference was found in CK5 expression in the circulation when blood was processed fresh or frozen in TRIzol.

### Conventional PCR

PCR for CK5 and GAPDH was performed on cDNA template using intron-spanning primers. All primers are listed 5′-3′. Primers used were hCK5spanf (CTTGTGGAGTGGGTGGCTAT), hCK5spanR (CCACTTGGTGTCCAGAACCT) (CK5 GeneID: 3852), GAPDHf (GGAGTCAACGGGTATTTGGT) and GAPDHr (GACAAGCTTCCCGTTCTCAG). Cycling conditions were 95°C for 5 min, 40 cycles of 95°C for 30 sec, 56°C for 30 sec, 72°C for 30 sec and followed by a 5 min extension step at 72°C.

### Taqman primer/probe design

Taqman primers and probe were designed using a web based tool from Genscript (https://www.genscript.com/ssl-bin/app/primer) with manual fine-tuning. The primer-probe set was selected so that the forward primer (TTCTTTGATGCGGAGCTGT) was positioned over an exon-exon junction. The reverse primer sequence is CATGGAGAGGACCACTGAGG. The resulting primer pair amplifies a 66 bp fragment. The Taqman probe is labeled at the 5′ end with a fluorescent dye (6-carboxy-fluorescein, FAM) and on the 3′ end with a quencher (6-carboxy-tetramethyl-rhodamine, TAMRA). Primers and probe were synthesized by IDT (Coralville, IA). The sequence of the probe is CCCAGATGCAGACGCATGTCTCTG. A Blast search (NCBI, NIH) showed no significant homology with other known genes in the database.

The housekeeping gene, glyceraldehyde 3-phosphate dehydrogenase (GAPDH), was obtained as a pre-designed VIC labeled qPCR assay from Applied Biosystems (4310884E) (Foster City, CA) and used according to the manufacturer's instructions.

### CK5 qPCR assay optimization and validation

Optimal primer/probe concentrations were determined by first varying primer concentrations against the maximum recommended concentration of probe (250 nM). Subsequently, the concentration of probe was titrated. The concentration at which, for both primers and probe, the ΔCt and ΔRn did not change with increasing concentrations was selected.

Singleplex and multiplex qPCR was then performed to determine the compatibility of the CK5 assay with the assay for the internal control GAPDH. No competition between assays was observed, thus permitting the use of multiplex qPCR.

To determine the sensitivity and reproducibility of the assay, serial dilutions of cDNA template representing 5 log steps were prepared and run in triplicate. There was very little variation between the Ct triplicate measurements with the average standard deviation being 0.12 and the maximum deviation being 0.6 for all triplicate readings whose average delta Ct was within the range of the assay detection limits (i.e. Ct<36).

### Taqman qPCR conditions

Real-time amplification and detection was performed with the ABI Step One Plus (Applied Biosystems) sequence detection system. Each reaction included 250 nM probe, 600 nM forward primer, 600 nM reverse primer, 7.5 µl Taqman Fast Universal PCR master mix (Applied Biosystems), nuclease free water, and, with the exception of the standard curve, 5 µl of cDNA corresponding to 50 ng of total RNA. Cycling conditions used were 20 sec at 95°C, and 40 cycles of 1 sec denaturation at 95°C and 20 sec annealing at 60°C.

### Quantification and statistics

The comparative Ct method for multiplex PCR was performed as outlined in the ABI PRISM 7700 Sequence Detection System User Bulletin #2 (available on the Applied Biosystems website: http://www3.appliedbiosystems.com). Each sample was run in triplicate and amplified for the gene of interest CK5 and the housekeeping gene GAPDH. Samples that required more than 30 cycles to reach the threshold cycle (Ct) for GAPDH were discarded as it possibly indicated poor cDNA quality. Otherwise, the triplicate Ct values for each sample were averaged resulting in mean Ct values for both CK5 and GAPDH. The CK5 Ct values were then standardized to the housekeeping gene by taking the difference: ΔCt = Ct[CK5]−Ct[GAPDH]. In order to compare ΔCt values between cases and controls, we averaged the ΔCt values among the control samples and used the avg(ΔCt in controls) as a reference or calibrator ΔΔCt = ΔCt[sample]−avg(ΔCt controls). The fold-change between each sample and the reference sample was calculated as 2^−ΔΔCt^ for negative ΔΔCt values (which indicated an increase in fold change) and −2^ΔΔCt^ for positive ΔΔCt values (indicating a fold change decrease)[13], [14].

Statistical analyses were primarily performed on ΔCt distributions, as they were closer to Gaussian than the fold change distributions, which were highly skewed. An exact Wilcoxon rank sum test was used to compare ΔCt distributions between transplant and control patients. Kendall rank correlation was used to test for associations between ΔCt values and other variables such as age and time. An exact Wilcoxon signed rank test was used to test for change in ΔCt values within patients over time. Mixed effects logistic regression models were used to study relationships between infection, rejection, and ΔCt. Scatterplots and box plots were helpful for visualizing the data and linear regression tools such as Cook's distance identified outliers. Bonferroni correction for multiple testing was considered in interpreting statistical significance. All analyses were performed using R, a free statistical program available at http://cran.r-project.org/.
